# Supplementary material for: Process evaluation of co-designed interventions to improve communication of positive newborn bloodspot screening results
Source: BMJ Open. 2021 Aug 27;11(8):e050773. doi: 10.1136/bmjopen-2021-050773 (PMC8404436; doi:10.1136/bmjopen-2021-050773)
Supplement: Supplementary data [file bmjopen-2021-050773supp001.pdf]

# Interview prompts related to success criteria for testing the co-designed interventions in routine practice

Laboratory pro forma

**Coherence:**

- Are the new standardised lab pro forma(s) easy to describe?
    - What is different compared to the existing pro forma(s)?
    - Do staff understand how to complete the new pro forma(s)?
    - Is the data presented in an accessible and easily understood manner for lab and clinical staff?
- Are the new standardised lab pro forma(s) distinct from other interventions?
    - Are the condition specific pro forma(s) needed?
- Do the new standardised lab pro forma(s) have a clear purpose?
    - Do lab and clinical staff think the new pro forma(s) reduce ambiguity?
    - Do lab and clinical staff think the new pro forma(s) improve communication of a positive NBS result?
    - Are the new pro forma(s) easy to complete (lab) and navigate (clinical teams)?
    - Do the new pro forma(s) collect all the required information?
- Do the new standardised lab pro forma(s) fit in with the overall goals of the organisation?
    - Are they comparable in terms of time needed for completion?

Cognitive participation

- Is it possible to recruit the staff from each study site? If <50% of staff approached, agree to participate, consider stopping in consultation with PPIAG.
- Are staff willing to invest the time required to implement the interventions into practice? If drop out rate ≥50% then consider stopping in consultation with PPIAG.

Collective action:

- Is the training required too time consuming to make this feasible in practice?
    - How long does the training take?
    - What resources are needed?
    - What approach/method is most appropriate?
- Are the interventions compatible with existing resources?
    - Does it take more or less time to complete the new pro forma(s)?
    - Are there any formatting issues?

Reflexive monitoring:

- Is implementation of the intervention sustainable?
    - Time needed to complete the new pro forma(s)
    - Training needs?
- Does the qualitative data imply any negative psychological sequelae from the implementation of the interventions? Any 'incidents' should be reported to and discussed with PPIAG.
    - Have any ‘missing’ data caused any issues?
- Are the interventions being implemented as planned (fidelity)? If not are the adaptations appropriate for local context?
    - Audit completion of the new proforma(s)

Communication checklists

Coherence:

- Is he purpose of the education checklists easy to describe?
    - What is different compared to the existing methods used for sharing information about a positive NBS result between lab and clinical staff?
    - Do staff understand how to complete the checklists?
    - Is the information on the checklists presented in an accessible and easily understood manner for lab and clinical staff?
    - Is the differentiation between screening and diagnostic clear (training requirements)?
- Are the checklists distinct from other interventions?
    - Are the checklists needed?
- Do the checklists have a clear purpose?
    - Do lab and clinical staff think the checklists reduce ambiguity?
    - Do lab and clinical staff think the checklists improve communication when a child receives a positive NBS result?
    - Are the new checklists easy to complete (lab) and navigate (clinical teams)?
    - Do the checklists collect all the required information?
    - Where should the checklists be held; medical notes, red book etc
- Do the new standardised lab pro forma(s) fit in with the overall goals of the organisation?
    - Are they comparable in terms of time needed for completion?
    - Do they facilitate effective communication between health professionals?

Cognitive participation

- Is it possible to recruit the staff from each study site? If <50% of staff approached, agree to participate, consider stopping in consultation with PPIAG.
- Are staff willing to invest the time required to implement the interventions into practice? If drop out rate ≥50% then consider stopping in consultation with PPIAG.

Collective action:

- Is the training required too time consuming to make this feasible in practice?
    - How long does the training take?
    - What resources are needed?
    - Differentiation between screening and diagnostic clear (training requirements)?
    - What approach/method is most appropriate?

- Are the interventions compatible with existing resources?
    - Does it take more or less time to complete the new checklists?
    - Are there any formatting issues?
    - Where is each part of the checklist stored? With parents (red book), clinical teams, medical notes?
    - Who has/needs access to the checklists?

Reflexive monitoring:

- Is implementation of the intervention sustainable?
    - Time needed to complete the new checklists
    - Training needs?
- Does the qualitative data imply any negative psychological sequelae from the implementation of the interventions? Any 'incidents' should be reported to and discussed with PPIAG.
    - Have any ‘missing’ data caused any issues?
- Are the interventions being implemented as planned (fidelity)? If not are the adaptations appropriate for local context?
    - Audit
        - Who is filling in each section of the checklists
        - Are they being completed satisfactorily?
        - Are any data being consistently completed incorrectly or not being completed?

Information provision

**Coherence:**

- Is the email and the identified information sources easy to describe?
    - What is different compared to the existing information provision?
    - Do staff understand how / when to use the email?
    - Are staff familiar with the information sources (web pages/app) provided?
    - Do parents find the information sources accessible and helpful?
    - Is the information in the email presented in an accessible and easily understood manner for clinical staff and parents?
- Are the email and resources distinct from other interventions?
    - Is the email needed?
    - Is it sufficient to provide website links or would it be better to have a link to a website where all the other resources are signposted?
    - Are website links sufficient or do clinicians parents indicate a preference for an App such as the metabolic app
- Does the information provision have a clear purpose?
    - Does the email reduce ambiguity from a staff and parental perspective?
    - Do clinical staff and parents think the email improves communication following a positive NBS result?
    - Is the email easy to complete (clinical staff) and interpret (parents)?
    - Do the websites/links/app meet parents' needs or is there a better way to present these information sources (e.g. a screening website or an app like the metabolic app)?
    - Does the email and the websites/app provide all the required information from a staff and parental perspective?
- Does the email fit in with the overall goals of the organisation?
    - Is it comparable in terms of time needed for completion?
    - Does it improve the parent experience of care?
    - Does it facilitate effective communication between health professionals and parents?
        - Does the email need to be translated into different languages if so, how many?

Cognitive participation

- Is it possible to recruit the staff from each study site? If <50% of staff approached, agree to participate, consider stopping in consultation with PPIAG.
- Are staff willing to invest the time required to implement the interventions into practice? If drop out rate ≥50% then consider stopping in consultation with PPIAG.

Collective action:

- Is the training required too time consuming to make this feasible in practice?
    - How long does the training take?
    - What resources are needed?
    - What approach/method is most appropriate?
- Are the interventions compatible with existing resources?
    - Who will complete and send the email?
    - Does it take more or less time to complete and send the email?
    - Are there any formatting issues?
    - Are parents able to access the links and have the resources to do this?

Reflexive monitoring:

- Is implementation of the intervention sustainable?
    - Time needed to complete the email?
    - Training needs?
    - Regular checking of links to ensure they continue to work (who and how often)
- Does the qualitative data imply any negative psychological sequelae from the implementation of the interventions? Any 'incidents' should be reported to and discussed with PPIAG.
    - Have any ‘missing’ data caused any issues?
- Are the interventions being implemented as planned (fidelity)? If not are the adaptations appropriate for local context?
    - Audit use of the email?
        - Who is sending the email?
        - Is the email being completed satisfactorily?
        - Are any data being consistently completed incorrectly or not being completed?
