# Supplementary material for: Process evaluation of co-designed interventions to improve communication of positive newborn bloodspot screening results
Source: BMJ Open. 2021 Aug 27;11(8):e050773. doi: 10.1136/bmjopen-2021-050773 (PMC8404436; doi:10.1136/bmjopen-2021-050773)
Supplement: Supplementary data [file bmjopen-2021-050773supp002.pdf]

Prompts for Parental Interviews

1. Please could you describe how you were told about X's positive newborn screening result?
2. How did this make you feel?
3. What support did you receive afterwards?
4. What support have you sought since?
5. Have you told family and friends about X's positive result? How did they respond?
6. Do you feel it has had any impact on your relationship with other family members or friends?
7. How do you feel now about X's positive newborn screening result?
8. Show interventions
    a. Ask about perceived purpose of each
    b. Ask about experienced or perceived advantages / disadvantages of each
9. Is there anything you think should or could be done differently?
10. Any other comments?
